# Mechanical Ventilation, Partial Pressure of Carbon Dioxide, Increased Fraction of Inspired Oxygen and the Increased Risk for Adverse Short-Term Outcomes in Cooled Asphyxiated Newborns

**DOI:** 10.3390/children8060430

**Published:** 2021-05-21

**Authors:** Stamatios Giannakis, Maria Ruhfus, Mona Markus, Anja Stein, Thomas Hoehn, Ursula Felderhoff-Mueser, Hemmen Sabir

**Affiliations:** 1 Department of General Pediatrics, Neonatology and Pediatric Cardiology, Faculty of Medicine, University Children’s Hospital, Heinrich-Heine-University Duesseldorf, 40225 Düsseldorf, Germany; stamatios.giannakis@gmail.com (S.G.); mona.markus@hotmail.de (M.M.); Thomas.Hoehn@med.uni-duesseldorf.de (T.H.); 2 Department of Pediatrics I/Neonatology, University Hospital Essen, University Duisburg Essen, 45147 Essen, Germany; maria.ruhfus@gmail.com (M.R.); Anja.Stein@uk-essen.de (A.S.); Ursula.Felderhoff@uk-essen.de (U.F.-M.); 3 Department of Neonatology and Pediatric Intensive Care, Children’s Hospital University of Bonn, 53127 Bonn, Germany; 4 German Centre for Neurodegenerative Diseases (DZNE), 53127 Bonn, Germany

**Keywords:** perinatal asphyxia, hypoxic–ischemic encephalopathy, therapeutic hypothermia, outcome, hypocapnia, hyperoxia, mechanical ventilation

## Abstract

Neonates treated with therapeutic hypothermia (TH) following perinatal asphyxia (PA) suffer a considerable rate of disability and mortality. Several risk factors associated with adverse outcomes have been identified. Mechanical ventilation might increase the risk for hyperoxia and hypocapnia in cooled newborns. We carried out a retrospective study in 71 asphyxiated cooled newborns. We analyzed the association of ventilation status and adverse short-term outcomes and investigated the effect of the former on pCO_2_ and oxygen delivery before, during and after TH. Death, abnormal findings on magnetic resonance imaging, and pathological amplitude-integrated electroencephalography traces were used to define short-term outcomes. The need for mechanical ventilation was significantly higher in the newborns with adverse outcomes (38% vs. 5.6%, *p* = 0.001). Compared to spontaneously breathing neonates, intubated newborns suffered from significantly more severe asphyxia, had significantly lower levels of mean minimum pCO_2_ over the first 6 and 72 h of life (HOL) (*p* = 0.03 and *p* = 0.01, respectively) and increased supply of inspired oxygen, which was, in turn, significantly higher in the newborns with adverse outcomes (*p* < 0.01). Intubated newborns with adverse short-term outcomes had lower levels of pCO_2_ over the first 36 HOL. In conclusion, need for mechanical ventilation was significantly higher in newborns with more severe asphyxia. In ventilated newborns, level of encephalopathy, lower pCO_2_ levels, and increased oxygen supplementation were significantly higher in the adverse short-term outcomes group. Ventilatory parameters need to be carefully monitored in cooled asphyxiated newborns.

## 1. Introduction

Despite all advances in perinatal care, perinatal asphyxia (PA) remains a serious condition that can lead to hypoxic–ischemic encephalopathy (HIE) in preterm and term neonates. HIE is associated with early neurodevelopmental impairment (e.g., seizures, childhood epilepsy, cerebral palsy) and high mortality rates [1]. To date, therapeutic hypothermia (TH) remains the only established treatment improving neurodevelopmental outcomes in near-term and term infants with moderate to severe HIE, although around 30% of the cooled infants, included in recent randomized controlled trials, died or suffered from long-term neurodevelopmental impairment [2].

In acute phase of brain injury due to PA, brain homeostasis is impaired due to abrupt reduction of cerebral blood flow (CBF), which reduces the sufficient delivery of oxygen and high-energy metabolites to neurons, leading to cell depolarization and cytotoxic edema (primary cell death) [3]. The acute insult is followed by a reperfusion phase with normalization of CBF and recovery of cell swelling and cerebral oxidative metabolism [4]. A latent phase with slightly reduced CBF [5,6] lasting over about six hours may then be followed by a secondary deterioration (6–16 h) with secondary cell death, seizures, and failure of oxidative metabolism [7,8,9].

The reduction of cerebral metabolic rate due to brain impairment following HIE leads to a reduction of CBF and consecutively to a reduction of the endogenous carbon dioxide (CO_2_)-production in the brain predisposing to hypocapnia [10]. Additionally, TH similarly reduces the cerebral metabolic rate and might as well predispose the asphyxiated newborn to hypocapnia [11]. The body’s physiological response to severe acidosis is an increase of ventilatory rate, also predisposing to hypocapnia. However, it is not known yet whether this “physiological hypocapnia” is beneficial or should be avoided in cooled asphyxiated newborns. Furthermore, frequently observed symptoms in asphyxiated newborns, such as delayed initiation of spontaneous breathing, respiratory depression, pulmonary hypertension, and seizures often necessitate mechanical ventilation (in >60% of asphyxiated term newborns), increasing the risk of high oxygen supplementation and hyperventilation with subsequent hypocapnia [12,13,14,15,16]. This high incidence of hypocapnia among asphyxiated neonates has been associated with adverse neurodevelopmental outcomes both in non-cooled [17,18] and cooled near-term and term asphyxiated newborns with HIE [18,19]. However, it is unclear whether all cooled asphyxiated newborns do require mechanical ventilation in all instances.

Lower levels of carbon dioxide have the potential to exacerbate the brain injury caused by PA by further reducing CBF due to cerebral vasoconstriction and by decreasing the oxygen supply due to the leftward shift of the oxygen–hemoglobin dissociation curve [20]. While the decreased CBF can be tolerated by healthy term infants, it could harm the previously injured brain, causing cell death due to the diminished oxygen delivery [21]. In pre-clinical animal models of HIE, hypocapnia results also in DNA fragmentation and membrane lipid peroxidation in mitochondria of cerebral cortical neurons and may result in apoptotic cell death [22].

Furthermore, a brief exposure to hyperoxia depletes the glial progenitor pool and impairs functional recovery of the brain after hypoxia–ischemia by increasing the oxidative stress and the cerebral inflammatory response [23]. Resuscitation with room air has been shown to reduce mortality in preterm and term newborns compared to resuscitation using 100% oxygen, highlighting the importance of oxygen toxicity [24].

The aim of this current study was to describe the rate of mechanical ventilation in cooled asphyxiated newborns with HIE in association with short-term outcomes. Furthermore, we aimed to correlate the rates of partial pressure of carbon dioxide (pCO_2_), it’s differential pressures (ΔpCO_2_) and the increased O_2_-supply in ventilated cooled asphyxiated newborns in comparison to non-ventilated cooled asphyxiated newborns. Additionally, we evaluated the association of low and high pCO_2_ and fraction of inspired oxygen (FiO_2_) levels and adverse short-term outcomes during the first days of life in the ventilated cooled asphyxiated newborns. Moreover, we compared the short-term outcomes between the intubated cooled asphyxiated newborns with pCO_2_ levels under 30 mmHg or FiO_2_ > 60% and the rest of the cohort.

## 2. Materials and Methods

### 2.1. Data Collection

We performed a retrospective data analysis. Data of cooled asphyxiated newborns from two level I (highest level of care) neonatal intensive care units (NICUs) were collected. Ethical approval was obtained from the local hospital ethic committees (19-8556-BO, 18-8191-BO, 2018-270-ProspDEuA, 2018-270-1). The infants were born between 2009 and 2018 and met the institutions’ inclusion criteria for therapeutic hypothermia:

A. Gestational age ≥ 36^+0^ weeks, ≤6 h of life (HOL) AND
B. Cord/arterial pH ≤ 7.0 OR base excess ≤ −16 in the first sixty minutes of life OR APGAR-Score ≤ 5 AND/OR continued need for resuscitation at 10 min of life (criteria of perinatal asphyxia) AND
C. Evidence of moderate-to-severe encephalopathy [25] OR
D. Abnormalities on amplitude-integrated electroencephalography (aEEG) for at least 20 min or clinical and/or aEEG-defined seizures [26]

Seventy-one (*n* = 71) term newborns were assigned to whole-body hypothermia (core temperature of 33–34 °C) for 72 h starting within the first 6 HOL followed by a rewarming phase at a rate of 0.5 °C per hour. The treatment protocols of the two NICUs were similar. Twenty-three (*n* = 23) newborns were born at (*n* = 14) or transferred to (*n* = 9) the first NICU (University Hospital Duesseldorf, Germany) and forty-eight (*n* = 48) newborns were born at (*n* = 29) or transferred to (*n* = 19) the second NICU (University Hospital Essen, Germany). Demographic details and clinical data were collected for each newborn according to medical notes including birth weight, gender, gestational age, birth place (inborn/outborn), APGAR scores at 5 and 10 min, first pH, bases excess and lactate before initiation of TH, need for resuscitation at birth, Sarnat HIE grade, initial temperature before starting TH, aEEG time to normal trace, onset of clinical or subclinical seizures, signs of meconium aspiration, minimum blood glucose levels in the first 6 and 72 HOL, need for mechanical ventilation, cumulative morphine dose needed until discharge from hospital, survival, need for inotropic support before and during TH, duration of O_2_-supplementation, and highest FiO_2_ levels within the first 6 and 72 HOL.

Additionally, we collected data regarding respiratory monitoring before and after the initiation of TH until the end of rewarming. This included arterial, capillary, and venous blood gases, which were corrected for temperature during TH, lowest pCO_2_ (minimum pCO_2_), highest pCO_2_ (maximum pCO_2_) and ΔpCO_2_ during the first 6 and 72 HOL as well as minimum and maximum pCO_2_ levels every 6 h after initiation of TH until 6 h after rewarming. The mode of ventilation (intubated vs. not intubated), duration of mechanical ventilation, as well as average and maximal oxygen supplementation (measured as mean and maximum FiO_2_ levels hourly) were also collected and analyzed. The indications for intubation and extubation were individually assessed from the neonatologists on duty and according to the International Liaison Committee on Resuscitation (ILCOR) recommendations for newborn resuscitation.

### 2.2. Outcome Definition

Adverse outcomes were defined as death or adverse magnetic resonance imaging (MRI) outcome. The original MRI-images (T1 and T2 weighted images) were evaluated by three independent individuals blinded to the clinical information. The basal ganglia/watershed score (BG/W score) developed by Barkovich defines MRI outcomes depending on severity and location of brain injury (1 = no injury, 2 = mild injury, 3 = moderate injury, 4 = severe injury) and discriminates accurately between asphyxiated newborns with good and poor neuromotor and cognitive outcomes at 3 and 12 months [27]. A recent study shows that this still holds true in the cooling-era with strong correlation of the BG/W score with long-term neurodevelopmental outcomes at 20–24 months of age [28]. For our study, the MRI outcomes were defined as good when the BG/W score was <2 and as adverse when the BG/W score was >2. MRI was available for 60/71 of the newborns in the cohort; 6 out of 9 newborns who died didn’t have one before death. For the other 5 newborns without MRI scans, we used aEEG as an outcome predictor, which has been shown to be a good prognostic outcome parameter and correlates well with MRI outcomes in cooled asphyxiated newborns [29,30,31].

In both NICUs single-use needle electrodes (positions equal to C3-P3, C4-P4 of a standard EEG) were applied to record biparietal aEEG signal. Continuous recording was established after postnatal clinical stabilization and before initiation of TH until the end of the rewarming phase (Brainz or Olympic Brainz Monitor, Natus, San Carlos, CA, USA). Three independent individuals blinded to the clinical information evaluated the aEEG traces retrospectively. The aEEG background pattern was classified as previously described [32], with continuous normal voltage (CNV) and discontinuous normal voltage (DNV) as normal patterns and burst suppression (BS), low voltage (LV), and flat trace as pathological patterns. Normal aEEG was defined as a time of under 48 h taken to reach a normal aEEG trace after the initiation of TH [32], and aEEG was scored as pathological when seizures were detected.

### 2.3. Data Analysis

SPSS 26 (SPSS, Chicago, IL, USA) was used for statistical analysis. Mann–Whitney was used to compare non-parametric data between two groups (intubated versus non-intubated and good versus adverse short-term outcomes in the intubated group). Descriptive data are presented as median and interquartile range (IQR) for continuous variables and as frequency distributions for categorical variables. Categorical variables were compared using a Chi-square test. In the intubated cooled newborns, multivariate analysis using stepwise binary logistic regression was performed with good or adverse outcomes as the dependent variable. Independent variables were APGAR scores at 5 and 10 min, first pH, severity of encephalopathy, seizures (yes/no), aEEG time to normal trace, lowest pCO_2_ (minimum pCO_2_), highest pCO_2_ (maximum pCO_2_) and ΔpCO_2_ during the first 6 and 72 HOL, and highest FiO_2_ levels within the first 6 and 72 HOL.

To avoid calculating the high levels of pCO_2_ in the cord gas and/or the first blood gases and the high oxygen supplementation during resuscitation, we used the trapezium rule to calculate the area under the curve (AUC) for pCO_2_ and FiO_2_ for the first HOL until the end of the rewarming phase [33]. *p* ≤ 0.05 was considered significant. Parts of the results from this cohort have already been published [31].

## 3. Results

Seventy-one (*n* = 71) cooled asphyxiated newborns ≥36 + 0 weeks of gestation were included in our study; thirty-four (47.9%) were males and thirty-seven (52.1%) were females. Fifty-three (74.6%) had a good and eighteen (25.4%) had an adverse short-term outcome (defined as death (*n* = 9) or an adverse MRI outcome or pathological aEEG when MRI was not available) despite TH. As previously shown, there is a strong correlation between aEEG and MRI outcome in our cohort [31].

Fifty-three (74.6%) of the newborns were intubated and mechanically ventilated based on the neonatologist’s discretion on duty. All of these newborns were intubated before initiation of TH within the first HOL and the mean (±SD) duration of mechanical ventilation was 91.8 (±86) h. The spontaneously breathing newborns (*n* = 18, 25.6%) were all respiratory-supported with continuous positive airway pressure (CPAP) or high-flow nasal cannula (HFNC). The need for mechanical ventilation was significantly correlated with adverse short-term outcomes (38% vs. 5.6%, *p* = 0.001).

Comparing the intubated and non-intubated cooled asphyxiated newborns, we found no significant differences between the two groups regarding birth weight, gestational age, first lactate level, time to initiation of therapeutic hypothermia, and time to target temperature. In our study neither lowest blood glucose levels within the first 72 HOL nor duration of morphine application impacted short-term outcomes (Table 1). However, we found that the APGAR scores at 5 and 10 min, as well as the cord or arterial pH and base excess values after birth were significantly lower in the intubated newborns who were treated with TH (*p* < 0.05). This is also reflected by the increased need for resuscitation in this group in comparison to the spontaneously breathing newborns (62.3% vs. 11.1%, *p* < 0.01). The first temperature measured after birth was significantly lower in newborns who needed mechanical ventilation (*p* = 0.01) and the severity of the Sarnat HIE grade was higher in the intubated group in comparison to the spontaneously breathing newborns (*p* < 0.01). We also found that the mechanically ventilated asphyxiated newborns had significantly lower levels of blood glucose in the first 6 HOL but these did not exceed the limits for hypoglycemia (<45 mg/dL). As expected, the need for ventilation required also significantly higher cumulative doses of morphine, and resulted in longer and higher oxygen supplementation (*p* < 0.05, Table 1). In addition, median (IQR) AUC mean and maximum FiO_2_ values were higher in mechanically ventilated newborns (0.21 (0.21–0.24) vs. 0.21 (0.21–0.21)%, and 0.23 (0.21–0.29) vs. 0.21 (0.21–0.215)%, *p* = 0.06 and *p* < 0.01, respectively).

We further analyzed the intubated group (*n* = 53) separately. We found that among cooled newborns who needed mechanical ventilation, the short-term outcomes were good in 36 (67.9%) vs. 17 (32.1%) with adverse outcomes. In intubated cooled newborns, the baseline characteristics (gender, gestational age, birth weight, birth place, first base excess and lactate levels, need for resuscitation at birth, meconium aspiration, initial temperature measured, as well as time to start TH and time to target temperature, lowest blood glucose levels at 6 and 72 HOL, cumulative dose of morphine, need for inotropes, duration of inspired oxygen and AUC mean and maximum FiO_2_ values) were not significantly different between the groups with normal vs. adverse outcomes (Table 2). Intubated newborns with good short-term outcomes had significantly higher APGAR scores at the 5th and 10th minute and higher cord or arterial pH values (*p* < 0.05). The severity of hypoxic–ischemic encephalopathy was significantly lower in the intubated newborns with good short-term outcomes (*p* < 0.001). Seizures and longer time (minutes) to normal trace of the amplitude-integrated EEG (73 (2–300) vs. 12 (1–23)) were significantly different in newborns with adverse short-term outcomes (*p* < 0.05). Intubated newborns with adverse outcomes received higher maximum FiO_2_ during the first 6 (*p* = 0.01) and 72 (*p* = 0.05) HOL.

**Figure 1:**
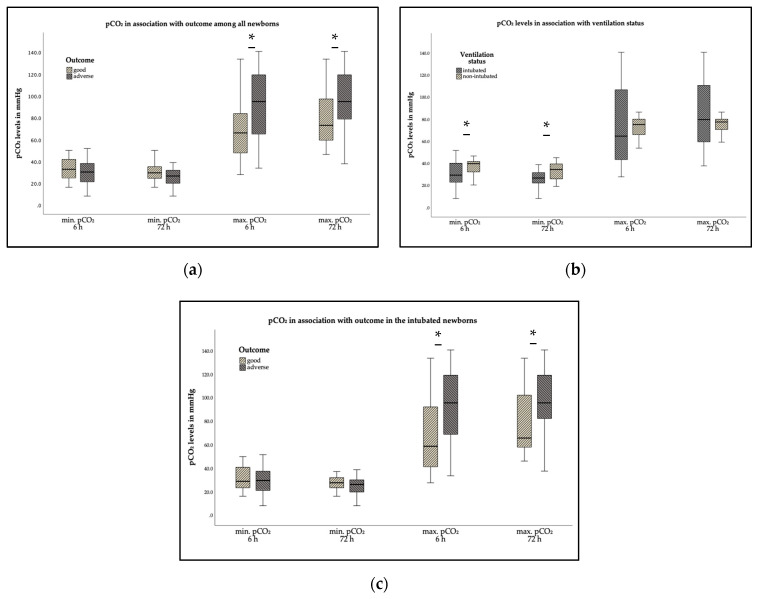
Box and whiskers plot representations. Minimum and maximum pCO_2_ over the first 6 and 72 h of life (HOL): (**a**) in association with adverse outcomes among all included cooled asphyxiated neonates, (**b**) in association with ventilation status, and (**c**) in association with adverse outcomes in the group of intubated newborns. * *p* < 0.05.

Evaluating significant differences of pCO_2_ levels and short-term outcomes among all neonates included in the study, we found that the mean (±SD) minimum pCO_2_ levels were lower within the first 6 and 72 HOL among newborns with adverse short-term outcomes (34.6 ± 12.6 vs. 31 ± 11.4 mmHg and 30.6 ± 9.3 vs. 26.4 ± 8.5 mmHg, *p* = 0.15 and *p* = 0.05, respectively) (Figure 1a). Interestingly, mean (±SD) maximum pCO_2_ within the first 6 and 72 HOL (92.7 ± 36 vs. 69.7 ± 27.9 mmHg and 97.1 ± 30.1 vs. 78.1 ± 22.8 mmHg) was significantly higher (*p* = 0.02 and *p* = 0.03, respectively) in the adverse short-term outcome group (Figure 1a). In addition, higher median ΔpCO_2_ (IQR), over the first 6 and 72 HOL was significantly associated with adverse short-term outcomes (31 (13.9–60.1) vs. 66.3 (39.9–98.7) mmHg and 41.0 (29.8–70.8) vs. 62.8 (44–97.6) mmHg), *p* < 0.01, respectively).

**Figure 2:**
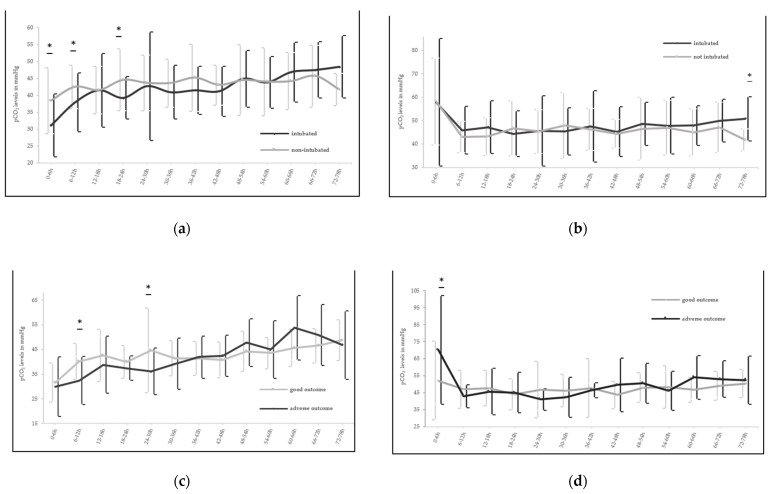
Significant differences between lowest and highest partial pressure of carbon dioxide (minimum and maximum pCO_2_) correlated with outcome and ventilation status. Temporal course of minimum (**a**) and maximum pCO_2_ (**b**) over the first 78 h after initiation of TH, examined every 6 h, in association with ventilation status and course of minimum (**c**) and maximum pCO_2_ (**d**) over the first 78 h after initiation of therapeutic hypothermia (TH) compared to outcomes in the subgroup of intubated newborns. Values are represented as mean ± standard deviation (SD), * *p* < 0.05.

Mean (±SD) minimum pCO_2_ was significantly lower in intubated newborns during the first 6 and 72 HOL vs. spontaneously breathing neonates (32.3 ± 13.4 vs. 37.4 ± 8.3 mmHg and 28.1 ± 9.4 vs. 33.4 ± 7.9 mmHg, *p* = 0.03 and *p* = 0.01, respectively, Figure 1b). This also holds true when analyzing mean minimum pCO_2_ every 6 h especially for the first 24 HOL (Figure 2a). Thirty (*n* = 30) intubated newborns had a pCO_2_ level under 30 mmHg at least once over the first 72 h with the lowest level being 8.4 mmHg in comparison to the non-ventilated newborns, where only five (*n* = 5) had a pCO_2_ under 30 mmHg with the lowest level being 19.4 mmHg. Intubated newborns with pCO_2_ <30 mmHg were more likely to have adverse short-term outcomes compared to the rest of cohort (*p* = 0.037), while all the spontaneously breathing newborns with pCO_2_ <30 mmHg had good short-term outcomes. Additionally, median AUC minimum pCO_2_ was significantly lower (*p* = 0.03) in mechanically ventilated newborns vs. spontaneously breathing newborns (42.6 (38.8–45.5) vs. 45.7 (41.4–50.6) mmHg).

**Figure 3:**
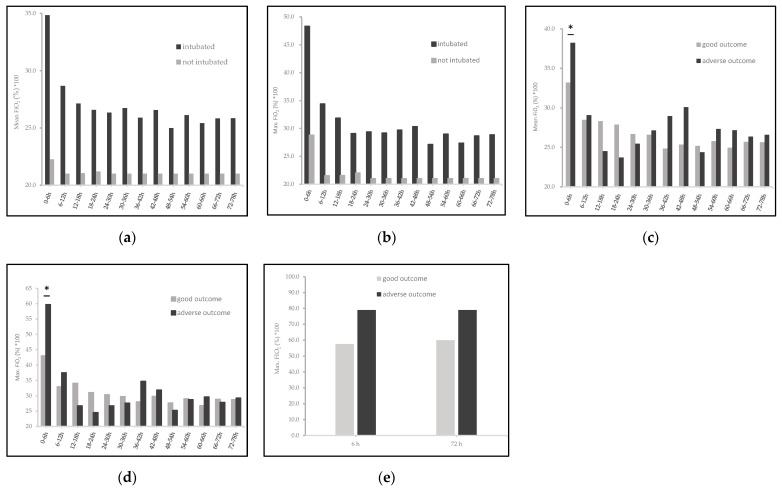
Significant differences of average and highest FiO_2_ (mean and maximum FiO_2_) with outcome and ventilation status. Maximum FiO_2_ over the first 6 and 72 HOL was directly associated with adverse outcomes in the whole study group (**e**), *p* < 0.05. Mechanically ventilated neonates had significantly higher needs of inspired oxygen, mean (**a**) and maximum (**b**) FiO_2_, during the whole period of TH, *p* < 0.05. Mean (**c**) and maximum (**d**) FiO_2_ were in total not significantly associated with adverse outcomes in the group of intubated newborns after the initiation of TH. Values are represented as mean (±SD), * *p* < 0.05.

The maximum pCO_2_ and AUC maximum pCO_2_ during the 72 h of TH were not significantly different within the two groups, except for the first 6 h of the rewarming phase, where the mean (±SD) maximum pCO_2_ levels were higher in the mechanically ventilated newborns (50.8 ± 9.4 vs. 41.7 ± 4.7 mmHg, *p* < 0.01, Figure 2b). Twenty-seven (*n* = 27) intubated cooled asphyxiated newborns had maximum pCO_2_ levels over 70 mmHg with the maximum pCO_2_ level being 140 mmHg during the first 72 HOL while only fourteen (*n* = 14) of the non-ventilated newborns had maximum pCO_2_ levels above 70 mmHg, with the highest level being 110 mmHg. Mechanical ventilation was significantly related to higher ΔpCO_2_ levels over the first 72 HOL (55.1 (30.3–79.4) vs. 43.7 (31.8–57.8), *p* = 0.03, Table 1). During the whole period of TH (including the 6 h of the rewarming phase) newborns who were intubated received significantly higher oxygen supplementation (measured as mean and maximum FiO_2_, *p* < 0.05) as seen in Figure 3a,b. Higher FiO_2_ within the first 6 and 72 HOL was also significantly different in the newborns with adverse short-term outcomes (*p* < 0.05, Figure 3e).

Comparing the short-term outcomes among ventilated newborns we found no significant association between mean (±SD) minimum pCO_2_ levels in the first 6 and 72 HOL and adverse outcomes (33.1 ± 14.3 vs. 30.5 ± 11.6 and 29.1 ± 9.8 vs. 25.6 ± 8.1 mmHg, *p* = 0.25 and *p* = 0.10 respectively, Figure 1c). However, lower levels were observed over the first 36 HOL and adverse outcomes were significantly higher in the newborns with lower mean (±SD) minimum pCO_2_ levels during the hours 6–12 (32.4 ± 9.8 vs. 40.2 ± 7.2 mmHg, *p* < 0.01) and 24–30 (36.2 ± 9.4 vs. 44.7 ± 17.2 mmHg) after initiating TH (Figure 2c). In this subgroup, we also found a significant difference of higher mean (±SD) maximum pCO_2_ levels during the first 6 (93.6 ± 37.1 vs. 66.8 ± 31.4 mmHg, *p* < 0.01) and 72 HOL (98.3 ± 30.8 vs. 78.1 ± 25.9 mmHg, *p* < 0.01) and adverse short-term outcomes (Figure 1c).

The result was strengthened from significantly higher median (IQR) AUC maximum pCO_2_ levels among intubated newborns with adverse short-term outcomes [50 (45.5–55.9) vs. 46.1 (43.6–50) mmHg, *p* < 0.05]. Higher mean (±SD) maximum pCO_2_ levels were significantly different in newborns with adverse outcomes only within the first 6 h after initiation of TH (70.2 ± 31.9 mmHg vs. 52.1 ± 23.1 mmHg, *p* < 0.05, Figure 2d). Comparing ΔpCO_2_ levels over the first 6 and 72 HOL we found significantly larger differences in the group of the mechanical ventilated asphyxiated newborns with unfavorable short-term outcomes (Table 2).

Oxygen supplementation among intubated newborns was not significantly different except for higher FiO_2_ levels in the first 6 HOL (*p* = 0.01, Table 2) and in the first 6 h after initiation of TH (*p* = 0.04, Figure 3c,d) in newborns with adverse short-term outcome. However, we found that for mechanically ventilated newborns with FiO_2_ above 0.60 in the first 6 (*n* = 30) and 72 (*n* = 33) HOL adverse short-term outcomes were significantly more likely when compared to neonates with lower oxygen supply (*p* < 0.05).

Regression analysis did not show any significant differences between the good or adverse outcome groups in the ventilated cooled newborns.

## 4. Discussion

The current study was performed to compare ventilated and non-ventilated cooled asphyxiated newborns in two large NICUs in Germany. We found that there was a significant difference between cooled asphyxiated infants who needed mechanical ventilation after birth and adverse short-term outcomes, in comparison with infants who were not intubated. The need for mechanical ventilation was significantly higher in newborns with more severe asphyxia. In ventilated newborns, level of encephalopathy, lower pCO_2_ levels within the first 24 h after birth and increased oxygen supplementation during the cooling period were significantly higher in the adverse short-term outcome group. In addition, higher maximum pCO_2_ levels and consequently higher ΔpCO_2_ levels were found in ventilated newborns with adverse short-term outcome.

Nadeem et al. showed in a small retrospective cohort study of cooled asphyxiated newborns that there was no association between pCO_2_ values and adverse outcome, although only 6 out of 52 infants maintained normocapnia in the first 72 h of life. As in our study severe hypocapnia (pCO_2_ < 20 mmHg) was documented only in ventilated infants [16]. We previously analyzed data from a cooling cohort in the UK, and also did not find an association between hypocapnia (defined as pCO_2_ < 30 mmHg) and adverse outcomes in a retrospective study of 61 cooled asphyxiated newborns [34]. In the current study, comparing pCO_2_ levels during the cooling period, we found that intubated newborns had significantly lower values of mean minimum pCO_2_ during the first 6 and 72 h after birth in comparison to spontaneously breathing infants (Figure 1b). The mean minimum pCO_2_ values were significantly lower in the intubated group, particularly in the first 12 HOL and at the end of the first day after initiation of TH (Figure 2a). Intubated newborns with adverse short-term outcomes had lower levels of mean minimum pCO_2_ over the first 36 HOL and especially during the hours 6–12 and 24–30 after initiation of TH. Additionally, intubated newborns with pCO_2_ levels under 30 mmHg had in general significantly more likely adverse short-term outcomes when compared to the rest of the cohort.

As mentioned in the introduction, the acute insult of PA with reduced CBF is followed by a reperfusion phase with restoration of cerebral circulation and (partial) recovery of the neuronal damage [3,4]. The latent phase is characterized by decreased metabolic rate and reduced CBF with increased tissue oxygenation [6], while the secondary deterioration correlates with an increase in CBF and metabolic demands due to the onset of seizures [5]. These frequent changes in the cerebral circulation highlight the importance of maintaining normocapnia, since CBF of the newborn is very sensitive to variations in pCO_2_ levels with a close to exponential relationship. A reduction in pCO_2_ of 1 kPa causes a reaction of 25–30% in CBF [35] and reduced CBF leads to cerebral cell death due to reduced cerebral oxygen supply [21]. In our study higher ΔpCO_2_ was associated with adverse short-term outcomes and was more frequently noticed among newborns who were mechanically ventilated.

To date, there are several reports showing an association of lower levels of pCO_2_ and adverse outcomes, mainly observed within the first HOL [15,16,17,18,19]. Klinger et al. first described the association between unfavorable outcomes and severe hypocapnia and/or severe hyperoxemia in the first 20 to 120 min after birth in non-cooled asphyxiated newborns [17]. Pappas et al. reported also that minimum pCO_2_ and cumulative pCO_2_ <35 mmHg over the first 12 h of life were significantly associated with unfavorable neurodevelopmental outcomes and higher risk of death, although in the subgroup of the infants treated with TH no significant association was documented [18]. The more recent study of Laporte et al., who evaluated pCO_2_ levels over a longer period (0–96 h of life), reported a significant association of brain injury in MRI in term cooled asphyxiated newborns with lower minimum pCO_2_ during the first 4 days of life and lower minimum pCO_2_ averaged over days 1–4 of life. As also shown in our current study, there was also a significant association of brain impairment with intubation and mechanical ventilation [15].

Our study and the study of Laporte et al. highlight the potential association of mechanical ventilation with adverse outcomes and lower pCO_2_ levels, suggesting close monitoring of ventilatory parameters and pCO_2_ changes during TH. The tendency to lower pCO_2_ levels is also enhanced by the impaired metabolism of the injured brain following perinatal asphyxia and also by the reduction of the metabolic rate with reduced carbon dioxide production in the brain due to TH [10]. Additionally, TH seems to be beneficial for lung mechanics, leading to increased tidal volume and minute ventilation [36,37], parameters that could lead to unintentional mechanical hyperventilation and consecutive hypocapnia. Hyperventilation is furthermore exacerbated by a strong respiratory drive to compensate for metabolic acidosis after asphyxia [38]. Although the spontaneously breathing newborns seem to compensate for lower pCO_2_ levels as an effect of the high respiratory drive, we believe that mechanically supported hypocapnia has a risk of leading to adverse outcome. Thus, it is essential to monitor ventilatory settings carefully and maintain normal levels of pCO_2_ during TH.

In our study, we found a significant difference of maximum pCO_2_ levels during the first 6 and 72 HOL and adverse short-term outcomes among all newborns included in our study and in the subgroup of intubated infants. This could be partially explained by the increase of metabolic demands associated with the greater seizure burden during the secondary deterioration 3–16 days after PA [5]. Until now there have been controversial reports about the effects of hypercapnia on the hypoxic–ischemic brain. Vannucci et al. were the first to report in two experimental studies that mild hypercapnia in immature rats with cerebral hypoxia–ischemia could protect from brain damage [39,40]. However, in the following years they showed that extreme hypercapnia could have an aggravating effect on hypoxic–ischemic brain damage [41]. In the already mentioned studies, which examined the levels of pCO_2_ in cooled asphyxiated newborns, only three studies compared the maximum levels of pCO_2_ in association with adverse outcomes without statistically significant results. Our study is the first to describe this correlation.

The deleterious effects of oxygen in asphyxiated term and preterm infants are also well-established. We and others have previously shown that an FiO_2_ above 0.40 within the first 6 HOL and severe hyperoxia (paO_2_ > 200 mmHg) during the first 20–120 min of life were associated with adverse outcomes in cooled asphyxiated newborns [17,34]. In the current study we show that during the whole period of cooling treatment, newborns, who had higher and longer needs of O_2_ supplementation, had significant worse short-term outcomes (Table 1 and Figure 3e). There were also significantly higher maximum levels of FiO_2_ in the newborns that needed mechanical ventilation (Figure 3a). In the subgroup of intubated newborns, the ones with higher maximum FiO2 over the first 6 and 72 HOL (Table 2) and especially during the first 6 h after initiation of TH (Figure 3c,d) had significantly more likely adverse short-term outcomes.

The observation mentioned above can probably be explained by the fact that the acute hypoxic–ischemic event, as well as the reperfusion/reoxygenation phase, are characterized by increased oxidative stress initiated through production of free radicals, leading to delayed cell death and neuronal loss [42,43,44]. Especially during reperfusion, the production of reactive oxygen species is proportional to oxygen concentration [43]. An additional exposure to hyperoxia, for example due to excessive oxygen delivery in the delivery room as seen in our cohort, might impair the functional recovery of the already compromised brain and lead to increased brain tissue damage due to induction of a cerebral proinflammatory response [23,45]. This is supported by Munkeby et al. who showed an increased brain damage in hypoxemic piglets after resuscitation with 100% oxygen in comparison with ambient air due to increased expression of matrix metalloproteinase (MMP) and production of extracellular glycerol [46]. Saugstad et al. showed later in a metanalysis a significant 31% reduction of neonatal mortality among term newborns resuscitated with room air rather than 100% O_2_. Interestingly, there was also a trend of reduction of the grade of HIE severity in newborns who received only room air during resuscitation [47]. In addition to these results, Dalen et al. highlighted that resuscitation with 100% oxygen counteracts the neuroprotective effect of TH in neonatal rats [48]. Although most of the studies compared delivery of 100% oxygen versus room air and not milder differences, as observed in our study, the findings assume that supply of oxygen, especially during the vulnerable phase of reperfusion/reoxygenation, should be used restrictively and should be carefully monitored during resuscitation and the first hours and days of life after PA. Since increased O_2_ supplementation is more likely to occur during mechanical ventilation, the routine application of the latter is once again critically questioned.

There are several limitations of this study. Blood-gas samples were a combination of arterial, capillary, and venous samples, since not every infant had an arterial line. This could probably underestimate the true degree of hypocapnia. Since our study was retrospective, we could not collect all desired data, such as partial pressure of oxygen and arterial oxygen saturation, mode of ventilation, ventilation frequencies and pressures, and definite cause or indications for intubation and extubation. These limitations could probably guide further prospective studies regarding respiratory support during therapeutic hypothermia. Additionally, blood gases were collected as clinically indicated and not at predefined times, so fluctuations of pCO_2_ between the samples could have been missed. The need for continuous pCO_2_ monitoring, as, for example, with transcutaneous CO_2_ or end-tidal CO_2_ monitoring, to better detect such fluctuations was highlighted by recent studies [15,49]. Technical difficulties and lack of systemic evaluation of these non-invasive techniques in cooled asphyxiated newborns remain unfortunately unsolved until now. Another limitation of this study is that we assessed the association of pCO_2_ and FiO_2_ levels with short-term outcomes and not with a standardized long-term outcome, such as the Bailey-Scales of infant development. The Barkovich MRI scoring is, however, an adequate scoring system, beside many others, which correlates well also with long-term outcomes until around 2 years of age [28]. Nevertheless, standardized long-term outcome assessments should be mandatory in all cooled asphyxiated newborns, as short-term assessments can never replace long-term neurodevelopmental outcome. Finally, our findings of low pCO_2_ levels in intubated newborns with adverse short-term outcomes might also have been supported by the increased severity of acidosis and encephalopathy in this group. Whether the physiological tendency of hyperventilation following severe acidosis does impair brain injury in cooled asphyxiated newborns, or should be tolerated is not known. However, we believe that if newborns are ventilated, ventilatory settings should be carefully adjusted and hyperventilation should be avoided. Our study had a small sample size, which limits the statistical power to analyze subgroups. Unfortunately, the German Neonatal Hypothermia Registry does not register data regarding ventilatory status and blood-gas parameters in cooled asphyxiated newborns. Furthermore, we found in our online survey on routine clinical practices of cooled asphyxiated newborns in Germany, that there is also wide heterogeneity in treatment practices in German NICUs [50]. Therefore, we aimed to analyze data of two large university NICUs (both highest level of care) in Germany with similar treatment protocols of perinatal asphyxia and hypoxic–ischemic encephalopathy.

## 5. Conclusions

Lower pCO_2_ levels and increased oxygen supply, which are well-known to be associated with adverse outcomes, were documented more frequently in intubated newborns in comparison to newborns without need of mechanical ventilation. Interestingly, mechanically ventilated newborns with lower pCO_2_ values had worse short-term outcomes compared to spontaneously breathing newborns with lower pCO_2_ values. Furthermore, higher ΔpCO_2_ levels, which were observed more frequently in intubated newborns, were significantly higher in newborns with adverse short-term outcomes. Comparing outcomes among intubated newborns, there were no significant associations of pCO_2_ and FiO_2_ values with adverse short-term outcomes. However, we have shown that the combination of mechanical ventilation and pCO_2_ < 30 mmHg or FiO_2_ >0.60 are significantly higher in newborns with adverse short-term outcomes, which may be also due to higher levels of encephalopathy in this group.

Mechanical ventilation in cooled asphyxiated newborns needs close monitoring to avoid hyperventilation and high ΔpCO_2_ levels. Additionally, oxygen supplementation should be restricted as much as possible to prevent additional oxidative stress in this sensitive group of newborns.

## Figures and Tables

**Table 1:** Descriptive data of the analyzed cohort according to ventilation status (mechanically ventilated or not) before the start of therapeutic hypothermia (TH). Data are presented as median and interquartile range (IQR).

Clinical Characteristics	Intubated (*n* = 53)	Non-Intubated (*n* = 18)	*p*-Value
Birth weight (g), median (IQR)	3265 (2845–3840)	3070 (2722.5–3545)	0.09
Male gender, *n* (%)	30 (56.6%)	4 (22.2%)	<0.01
Gestational age in weeks, median (IQR)	39^+6^ (37^+5^–40^+4^)	38^+6^ (37^+1^–39^+6^)	0.12
APGAR score			
5 min, median (min, max)	4 (0–10)	5 (2–9)	0.03
10 min, median (min, max)	6 (0–10)	7 (4–10)	**<0.01**
First pH, median (IQR)	6.81 (6.68–6.93)	6.93 (6.85–6.98)	**<0.01**
First base excess (mmol/L), median (IQR)	22.15 (16.6–27)	18 (14.2–22)	**<0.01**
First lactate level (mmol/L), median (IQR)	12.7 (8.7–17)	10.95 (8.52–13.08)	0.11
HIE grade before cooling (*n* = mild, *n* = moderate, *n* = severe)	7, 22, 21	11, 5, 0	**<0.01**
Inborn, *n* (%)	29 (54.7%)	14 (77.8%)	**0.03**
Resuscitation at birth, *n* (%)	33 (62.3%)	2 (11.1%)	**<0.01**
Short-term adverse outcome	17 (32.1%)	1 (5.6%)	**<0.01**
Death, *n* (%)	9 (17.0%)	0 (0%)	**<0.01**
Initial temperature (℃) before start of TH, median (IQR)	35.5 (34.2–36.9)	36.1 (35.8–36.4)	**0.01**
Time (minutes) until start ofTH, median (IQR)	37.5 (10–73.7)	30 (10–105)	0.50
Time (minutes) to target temperature, median (IQR)	120 (60–170)	120 (60–127.5)	0.37
EEG time (minutes) to normal trace, median (IQR)	13 (1–57)	1 (1–4.5)	**<0.01**
Lowest blood glucose levels (mg/dL)			
first 6 HOL, median (IQR)	81 (60–127)	68 (62.75–77.5)	**0.04**
first 72 HOL, median (IQR)	63 (48–77.5)	60 (48.5–68.5)	0.08
Morphine			
duration (hours), median (IQR)	72 (65–92)	72 (64–76)	0.09
cumulative dose (µg/kg/d), median (IQR)	0.6 (0.3–1.15)	0.25 (0.18–0.42)	**<0.01**
Inotropic support, *n* (%)	33 (62.3%)	4 (22.2%)	**<0.01**
Oxygen supplementation			
duration (minutes), median (IQR)	24 (8–102)	25 (0–3.25)	**<0.01**
highest FiO_2_(%) × 100			
first 6 HOL, median (IQR)	80 (40–100)	21 (21–58)	**0.01**
first 72 HOL, median (IQR)	80 (48–100)	21 (21–58)	**<0.01**
Area under the curve (AUC) FiO_2_(%) × 100 over 78 h			
maximum FiO_2_, median (IQR)	23.6 (21.3–29.6)	21 (21–21.5)	**<0.01**
mean FiO_2_, median (IQR)	21.6 (21–24)	21 (21–21)	0.06
AUC pCO_2_ over 78 h			
maximum pCO_2_, median (IQR)	42.6 (38.8–45.5)	45.7 (41.4–50.6)	**0.03**
minimum pCO_2_, median (IQR)	46.7 (44.1–53.5)	47.5 (41.7–54.9)	0.29
ΔpCO_2_ in mmHg			
first 6 HOL, median (IQR)	44.0 (15–74.3)	37.4 (27.7–58.75)	0.14
first 72 HOL, median (IQR)	55.1 (30.3–79.4)	43.7 (31.8–57.8)	**0.03**

**Table 2:** Descriptive data of the intubated cooled asphyxiated neonates according to short-term outcome. Data are presented as median and IQR.

Clinical Characteristics	Good Short-Term Outcomes (*n* = 36)	Adverse Short-Term Outcomes * (*n* = 17)	*p*-Value
Birth weight (g), median (IQR)	3212.5 (2827.5–3855)	3300 (2775–3827.5)	0.40
Male gender, ***n*** (%)	22 (61.1%)	8 (47.1%)	0.18
Gestational age (weeks), median (IQR)	38+6 (37 + 1–40 + 4)	40+2 (38 + 5–40 + 6)	0.07
APGAR score			
5 min, median (min, max)	5 (0–10)	2 (0–6)	**<0.01**
10 min, median (min, max	7 (1–10)	4 (0–7)	**<0.01**
First pH, median (IQR)	6.85 (6.78–6.95)	6.8 (6.6–6.92)	**0.03**
First base excess (mmol/L), median (IQR)	21.8 (16.6–25.1)	23 (16.35–29.6)	0.27
First lactate level (mmol/L), median (IQR)	12.2 (7.65–17)	13.5 (11.2–19)	0.11
HIE grade before cooling (***n*** = mild, ***n*** = moderate, ***n*** = severe)	7, 18, 9	0, 4, 12	**<0.01**
Inborn, ***n*** (%)	18 (50.0%)	11 (64.7%)	0.16
Resuscitation at birth, ***n*** (%)	20 (55.6%)	13 (76.5%)	0.08
Meconium aspiration, ***n*** (%)	8 (22.2%)	8 (47.1%)	0.15
Seizures, ***n*** (%)	15 (41.7%)	14 (82.4)	**<0.01**
Initial temperature (℃) before Start TH, median (IQR)	35.65 (34.1–37)	35.15 (34.3–36.5)	0.46
Time (minutes) until Start TH, median (IQR)	45 (10–77.5)	20 (10–71.3)	0.14
Time (minutes) to target temperature, median (IQR)	105 (52.5–150)	135 (60–183.5)	0.24
EEG time (minutes) to normal trace, median (IQR)	12 (1–23)	73 (2–300)	**<0.01**
Minimum blood glucose levels (mg/dL)			
first 6 HOL median (IQR)	77 (50–113)	86 (72–155)	0.25
first 72 HOL, median (IQR)	60 (45–79)	66 (50.3–74.8)	0.18
Morphine			
duration (hours), median (IQR)	79.5 (72–96)	67 (35–72)	**<0.01**
Cumulative dose (µg/kg/d), median (IQR)	0.61 (0.34–1.47)	0.42 (0.25–0.8)	0.43
Inotropic support, ***n*** (%)	22 (61.1%)	11 (64.7%)	0.39
Oxygen supplementation			
duration (minutes), median (IQR)	31.5 (3.75–106.5)	24 (11–88)	0.26
highest FiO_2_(%) × 100			
first 6 HOL, median (IQR)	60 (30–100)	80 (30–100)	**0.01**
first 72 HOL, median (IQR)	95 (68–100)	90 (67–100)	0.05
Area under the curve (AUC) FiO_2_(%) × 100 over 78 h			
maximum FiO_2_, median (IQR)	22.6 (21.2–28.3)	26.2 (23.6–37.2)	0.46
mean FiO_2_, median (IQR)	21.8 (21–23.9)	24.3 (21.6–32.8)	0.37
AUC pCO_2_ (mmHg) over 78 h			
maximum pCO_2_, median (IQR)	46.1 (43.6–50)	50 (45.5–55.9)	**0.049**
minimum pCO_2_, median (IQR)	42.6 (39.6–45.5)	42.4 (37.1–46.0)	0.20
ΔpCO_2_ in mmHg			
first 6 HOL, median (IQR)	25.9 (14.4–63.9)	70.2 (40.9–101.3)	**<0.01**
first 72 HOL, median (IQR)	41.0 (29.2–76.8)	66.3 (55.1–98.7)	**0.01**

* Adverse short-term outcomes defined as death (***n*** = 9) or severe brain damage using magnetic resonance imaging (MRI, ***n*** = 7) or pathological amplitude-integrated EEG (aEEG) traces (***n*** = 1) when MRI-data not available.

## Data Availability

Data can be accessed and is available from the authors.

## References

[1] Kurinczuk J.J., White-Koning M., Badawi N. (2010). Epidemiology of neonatal encephalopathy and hypoxic–ischaemic encephalopathy. Early Hum. Dev..

[2] Shankaran S., Laptook A.R., Pappas A., McDonald S.A., Das A., Tyson J.E., Poindexter B.B., Schibler K., Bell E.F., Heyne R.J. (2017). Effect of Depth and Duration of Cooling on Death or Disability at Age 18 Months Among Neonates With Hypoxic-Ischemic Encephalopathy: A Randomized Clinical Trial. JAMA.

[3] Tan W.K.M., Williams C.E., During M.J., Mallard C.E., Gunning M.I., Gunn A., Gluckman P.D. (1996). Accumulation of Cytotoxins During the Development of Seizures and Edema after Hypoxic-Ischemic Injury in Late Gestation Fetal Sheep. Pediatr. Res..

[4] Williams C.E., Gunn A., Gluckman P.D. (1991). Time course of intracellular edema and epileptiform activity following prenatal cerebral ischemia in sheep. Stroke.

[5] Gunn A.J., Gunn T.R., De Haan H.H., Williams C.E., Gluckman P.D. (1997). Dramatic neuronal rescue with prolonged selective head cooling after ischemia in fetal lambs. J. Clin. Investig..

[6] Jensen E.C., Bennet L., Hunter C.J., Power G.C., Gunn A.J. (2006). Post-hypoxic hypoperfusion is associated with suppression of cerebral metabolism and increased tissue oxygenation in near-term fetal sheep. J. Physiol..

[7] Roth S.C., Edwards A.D., Cady E.B., Delpy D.T., Wyatt J.S., Azzopardi D., Baudin J., Townsend J., Stewart A.L., Reynolds E.O.R. (2008). Relation between cerebral oxidative metabolism following birth asphyxia, and neurodevelopmental outcome and brain growth at one year. Dev. Med. Child Neurol..

[8] Roth S.C., Baudin J., Cady E., Johal K., Townsend J.P., Wyatt J.S., Reynolds E.O.R., Stewart A.L. (2008). Relation of deranged neonatal cerebral oxidative metabolism with neurodevelopmental outcome and head circumference at 4 years. Dev. Med. Child Neurol..

[9] Lorek A., Takei Y., Cady E.B., Wyatt J.S., Penrice J., Edwards A.D., Peebles D., Wylezinska M., Owen-Reece H., Kirkbride V. (1994). Delayed (“Secondary”) Cerebral Energy Failure after Acute Hypoxia-Ischemia in the Newborn Piglet: Continuous 48-Hour Studies by Phosphorus Magnetic Resonance Spectroscopy. Pediatr. Res..

[10] Yenari M.A., Han H.S. (2012). Neuroprotective mechanisms of hypothermia in brain ischaemia. Nat. Rev. Neurosci..

[11] Wood T., Thoresen M. (2015). Physiological responses to hypothermia. Semin. Fetal Neonatal Med..

[12] Rainaldi M.A., Perlman J.M. (2016). Pathophysiology of Birth Asphyxia. Clin. Perinatol..

[13] Morton S.U., Brodsky D. (2016). Fetal Physiology and the Transition to Extrauterine Life. Clin. Perinatol..

[14] Lapointe A., Barrington K.J. (2011). Pulmonary hypertension and the asphyxiated newborn. J. Pediatr..

[15] Laporte M.A.L., Wang H., Sanon P.-N., Vargas S.B., Maluorni J., Rampakakis E., Wintermark P. (2017). Association between hypocapnia and ventilation during the first days of life and brain injury in asphyxiated newborns treated with hypothermia. J. Matern. Neonatal Med..

[16] Nadeem M., Murray D., Boylan G., Dempsey E.M., Ryan C.A. (2009). Blood Carbon Dioxide Levels and Adverse Outcome in Neonatal Hypoxic-Ischemic Encephalopathy. Am. J. Perinatol..

[17] Klinger G., Beyene J., Shah P., Perlman M. (2005). Do hyperoxaemia and hypocapnia add to the risk of brain injury after intrapartum asphyxia?. Arch. Dis. Child. Fetal Neonatal Ed..

[18] Pappas A., Shankaran S., Laptook A.R., Langer J.C., Bara R., Ehrenkranz R.A., Goldberg R.N., Das A., Higgins R.D., Tyson J.E. (2011). Hypocarbia and Adverse Outcome in Neonatal Hypoxic-Ischemic Encephalopathy. J. Pediatr..

[19] Lingappan K., Kaiser J.R., Srinivasan C., Gunn A., on behalf of the CoolCap Study Group (2016). Relationship between PCO_2_ and unfavorable outcome in infants with moderate-to-severe hypoxic ischemic encephalopathy. Pediatr. Res..

[20] Laffey J.G., Kavanagh B.P. (2002). Hypocapnia. N. Engl. J. Med..

[21] Victor S., Appleton R.E., Beirne M., Marson A.G., Weindling A.M. (2005). Effect of carbon dioxide on background cerebral electrical activity and fractional oxygen extraction in very low birth weight infants just after birth. Pediatr. Res..

[22] Pirot A.L., Fritz K.I., Ashraf Q.M., Mishra O.P., Delivoria-Papadopoulos M. (2007). Effects of Severe Hypocapnia on Expression of Bax and Bcl-2 Proteins, DNA Fragmentation, and Membrane Peroxidation Products in Cerebral Cortical Mitochondria of Newborn Piglets. Neonatology.

[23] Koch J.D., Miles D.K., Gilley J.A., Yang C.-P., Kernie S.G. (2008). Brief Exposure to Hyperoxia Depletes the Glial Progenitor Pool and Impairs Functional Recovery after Hypoxic-Ischemic Brain Injury. Br. J. Pharmacol..

[24] Davis P.G., Tan A., O’Donnell C.P., Schulze A. (2004). Resuscitation of newborn infants with 100% oxygen or air: A systematic review and meta-analysis. Lancet.

[25] Sarnat H.B., Sarnat M.S. (1976). Neonatal encephalopathy following fetal distress. A clinical and electroencephalographic study. Arch. Neurol..

[26] Al Naqeeb N., Edwards A.D., Cowan F.M., Azzopardi D. (1999). Assessment of Neonatal Encephalopathy by Amplitude-integrated Electroencephalography. Pediatrics.

[27] Barkovich A.J., Hajnal B.L., Vigneron D., Sola A., Partridge J.C., Allen F., Ferriero D.M. (1998). Prediction of neuromotor outcome in perinatal asphyxia: Evaluation of MR scoring systems. Am. J. Neuroradiol..

[28] Al Amrani F., Marcovitz J., Sanon P.-N., Khairy M., Saint-Martin C., Shevell M., Wintermark P. (2018). Prediction of outcome in asphyxiated newborns treated with hypothermia: Is a MRI scoring system described before the cooling era still useful?. Eur. J. Paediatr. Neurol..

[29] Thoresen M., Hellström-Westas L., Liu X., De Vries L.S. (2010). Effect of Hypothermia on Amplitude-Integrated Electroencephalogram in Infants With Asphyxia. Pediatrics.

[30] Sarkar S., Barks J.D., Donn S.M. (2008). Should amplitude-integrated electroencephalography be used to identify infants suitable for hypothermic neuroprotection?. J. Perinatol..

[31] Ruhfus M., Giannakis S., Markus M., Stein A., Hoehn T., Felderhoff-Mueser U., Sabir H. (2021). Association of Routinely Measured Proinflammatory Biomarkers With Abnormal MRI Findings in Asphyxiated Neonates Undergoing Therapeutic Hypothermia. Front. Pediatr..

[32] Toet M.C., Hellström-Westas L., Groenendaal F., Eken P., De Vries L.S. (1999). Amplitude integrated EEG 3 and 6 hours after birth in full term neonates with hypoxic-ischaemic encephalopathy. Arch. Dis. Child. Fetal Neonatal Ed..

[33] Reinhardt F., Soeder H., Falk G. (1974). DTV-Atlas zur Mathematik: Taf. u. Texte. Orig.-Ausg. Ed.

[34] Sabir H., Jary S., Tooley J., Liu X., Thoresen M. (2012). Increased Inspired Oxygen in the First Hours of Life is Associated with Adverse Outcome in Newborns Treated for Perinatal Asphyxia with Therapeutic Hypothermia. J. Pediatr..

[35] Greisen G. (2005). Autoregulation of cerebral blood flow in newborn babies. Early Hum. Dev..

[36] Cavallaro G., Filippi L., Cristofori G., Colnaghi M., Ramenghi L., Agazzani E., Ronchi A., Florini P., Mosca F. (2011). Does pulmonary function change during whole-body deep hypothermia?. Arch. Dis. Child. Fetal Neonatal Ed..

[37] Dassios T., Austin T. (2014). Respiratory function parameters in ventilated newborn infants undergoing whole body hypothermia. Acta Paediatr..

[38] Thoresen M. (2008). Supportive care during neuroprotective hypothermia in the term newborn: Adverse effects and their prevention. Clin. Perinatol..

[39] Vannucci R.C., Towfighi J., Heitjan D.F., Brucklacher R.M. (1995). Carbon dioxide protects the perinatal brain from hypoxic-ischemic damage: An experimental study in the immature rat. Pediatrics.

[40] Vannucci R.C., Brucklacher R.M., Vannucci S.J. (1997). Effect of carbon dioxide on cerebral metabolism during hypoxia-ischemia in the immature rat. Pediatr. Res..

[41] Vannucci R.C., Towfighi J., Brucklacher R.M., Vannucci S.J. (2001). Effect of Extreme Hypercapnia on Hypoxic-Ischemic Brain Damage in the Immature Rat. Pediatr. Res..

[42] Bracci R., Perrone S., Buonocore G. (2001). Red blood cell involvement in fetal/neonatal hypoxia. Biol. Neonate.

[43] Saugstad O.D., Aasen A.O. (1980). Plasma hypoxanthine concentrations in pigs. A prognostic aid in hypoxia. Eur. Surg. Res..

[44] Núnez A., Benavente I., Blanco D., Boix H., Cabañas F., Chaffanel M., Fernández-Colomer B., Fernández-Lorenzo J.R., Loureiro B., Moral M.T. (2018). Oxidative stress in perinatal asphyxia and hypoxic-ischaemic encephalopathy. An. Pediatría.

[45] Markus T., Hansson S., Amer-Wåhlin I., Hellström-Westas L., Saugstad O.D., Ley D. (2007). Cerebral Inflammatory Response After Fetal Asphyxia and Hyperoxic Resuscitation in Newborn Sheep. Pediatr. Res..

[46] Munkeby B.H., Børke W.B., Bjørnland K., Sikkeland L.I.B., Borge G.I.A., Halvorsen B., Saugstad O.D., Oslash B.W.B. (2004). Resuscitation with 100% O_2_ increases cerebral injury in hypoxemic piglets. Pediatr. Res..

[47] Saugstad O.D., Ramji S., Soll R.F., Vento M. (2008). Resuscitation of Newborn Infants with 21% or 100% Oxygen: An Updated Systematic Review and Meta-Analysis. Neonatology.

[48] Dalen M.L., Liu X., Elstad M., Løberg E.M., Saugstad O.D., Rootwelt T., Thoresen M. (2012). Resuscitation with 100% oxygen increases injury and counteracts the neuroprotective effect of therapeutic hypothermia in the neonatal rat. Pediatr. Res..

[49] Szakmar E., Jermendy A., El-Dib M. (2019). Respiratory management during therapeutic hypothermia for hypoxic-ischemic encephalopathy. J. Perinatol..

[50] Giannakis S., Ruhfus M., Rüdiger M., Sabir H., Network T.G.N.H., Network G.N.H. (2019). Hospital survey showed wide variations in therapeutic hypothermia for neonates in Germany. Acta Paediatr..

